# Bullous Dermatitis Artefacta in a 17 Year-old Girl Induced by a Native Herb

**DOI:** 10.5812/ircmj.8886

**Published:** 2013-09-05

**Authors:** Mina Zarei, Mohammad Kamali, Reza Bidaki

**Affiliations:** 1 Department of Dermatology and Cutaneous Surgery, University of Miami, Miller School of Medicine, Miami, USA; 2 Department of Psychiatry, Rafsanjan University of Medical Sciences, Rafsanjan, IR Iran; 3 Department of Psychiatry, Rafsanjan University of Medical Sciences, Rafsanjan, IR Iran

**Keywords:** Dermatitis, Factitious Disorders, Bollous lesions

## Abstract

Dermatitis artifacta is a factitious dermatological disorder with many forms of presentation of self inflicted skin lesions in any part of the body. Dermatitis artefacta is a rare and difficult condition for diagnosis and treatment mostly because of the patient's denial. The liaison among primary care physicians, psychiatrists and dermatologists can be important in the management of these patients. In this report we describe a 17-year-old girl with dermatitis artefacta which was presented as bullous lesions on her face induced with a native herb combining with fake headaches.

## 1. Introduction

Dermatitis artifacta is a factitious dermatological disorder with many forms of presentation of self inflicted skin lesions in any part of the body (1). In dermatology, there are various mechanical injuries which caused with pressures, friction, occlusion, biting, cutting, stabbing, thermal burns or self-inflicted infections with wound-healing impairment, abscesses, mutilations or damages with acids and other toxic to the skin (2). Dermatitis artefacta is a rare and difficult condition for diagnosis and treatment, with the highest incidence of onset in late adolescence to early adulthood. Most patients are young women who have a personality disorder; borderline features are common and patient's denial of psychological distress makes management and treatment difficult (3). Triggering factors are psychiatric disorders or specific stress situations (4). Diagnosis of the disorder is often time-consuming and complicated process that precedes acceptance of the underlying pathology by both the therapist and patient and is often concluded after rigorous and repeated investigation (1, 4).

Recovery period is more associated with change in life situation compare to treatment period. The upper dose range of selective serotonin reuptake inhibitors (SSRIs), or low dose atypical antipsychotic agents, may be effective (5, 6).

## 2. Case Presentation

A 17-year-old girl came to the Neurology clinic with her family with the chief complaint of a chronic headache. She had pulsatile, mostly generalized headaches since 5 years ago. The patient had experienced these headaches twice a week or sometimes every two or three months. She didn't have any nausea, vomiting, dizziness or paresis of extremities during the episodes of headache. She was living in a village with her family with very poor socio-economical situation. She quit school after the sixth grade, was single and wasn't work. She had four siblings and was the last child of the family. There were no abnormal medical or psychological histories in her family. Based on the information that she and her family gave to the physicians she had no physical or psychological abnormalities before. The only positive physical finding at the neurology clinic was bullous and erythematic lesions on her forehead, cheeks and eyelids. The bullouses were multiple, tens with 2 to 3 cm diameter, symmetrical on the frontal and zygomatical regions of her face without involving a specific dermatome. The lesions had no pain, purities or discharge. There were no lesions elsewhere on her body.

The patient was admitted to the neurology ward for more evaluation and with the primary diagnosis of migraine attack. During hospitalization she was wxamined and all of her laboratory tests including reumatological tests and imaging were normal. EMG- NCV test was also performed and the result showed no abnormality. Based on her complaint, Giant Cell Arthritis examination also was done which resulted normal ESR and normal temporal artery biopsy. The patient was visited by an ophthalmologist and with the diagnosis of bilateral belepharitis and conjunctivitis Tetracycline ointment and eye drop. An ophthalmologist diagnosed bilateral belepharitis and conjunctivitis Tetracycline ointment and eye drop about the patient. Chloramphenicol eye drop and artificial tear drop was administered for her. Also, according to the consultation with a dermatologist, contact dermatitis was diagnosed for her and the treatment started with Hydrocortisone cream and 20 mg/day prednisone. The result of her skin biopsy revealed dermatological disorders like bullous pemphigoid or pemphigus vulgaris. With all these treatments she still had the headaches and new lesions were appeared on her face. Therefore, psychiatric consultation and interview by an expert psychiatrist was done for her.

During the interview, she was so shy and rolled her hands and crossed her legs together. She spoke with a very low tone voice and didn't like to talk with the doctor. She was so sensitive and irritable with a slight tremor on her cheeks and lips and was crying most of the time. At the first session she wasn't cooperative with the interviewer but in the next sessions she was more cooperative and reliable than before. The patient explained that she had committed suicide once before because of her nephew death about five years ago. Depakin was prescribed for her to cure her headache and depression. She attempted another suicide by taking poison a few months ago after an argument with her sister. She said that she had been physically and emotionally but not sexually abused by her brother, sister, father and uncle several times. She was so upset regarding arguments among her family members. She had no control on her life in the family; only men had the right to make decisions. She also had lack of kindness and attention from her family members and was so nervous and depressed. Because of her emotional distress and irritability she used a native herbal medication without any professional advice. When she started to use the herb her headaches have been started and the bullouses appeared on her skin. These problems resulted attentions from her family to her what she experienced before rarely. When she understood the herb was the reason for disorder, she started to fake the headaches without taking the herb and using the herb locally to induce the skin bullouses.

Eventually, psychiatric interviews specified that she had a sensitive and dependent personality. After several examinations and considering all her medical problems Major Depressive Disorder (MDD) and factitious disorder were diagnosed based on the DSM-IV criteria and treatment was started. Citalopram tablet 20 mg per day and Clonazepam 0.5 mg every night was prescribed for her. The patient and her family were registered for psychological therapy sessions. She also underwent ventilation therapy for one hour in three separate sessions. The skin bullouses were treated by occlusion techniques. She has been followed for six months with monthly visits at the psychiatry clinic. She didn't complaint from headaches anymore and the lesions were about to heal completely. Family training was obviously effective for her therapy. The patient gave the informed consent to report her case but without revealing her name or any picture of her lesions even with covering her eyes.

## 3. Conclusion

Dermatitis artefacta is a rare condition and most patients are young women who deny their problem like this case (3). Other psychiatric disorders are also common and include major depression, generalized anxiety disorder and phobic states like our patient who was suffering from major depression (7). Treatment with selective serotonin reuptake inhibitors (SSRIs), or low dose atypical antipsychotic agents was effective and this patient had an acceptable response to citalopram and clonazepam (6).

Cooperation among dermatologists, psychiatrists and the patient's family members is required for ensuring a favorable prognosis. These patients often resist psychiatric referral, and the liaison among primary care physicians; psychiatrists and dermatologists can be important in the management of these patients. Unfortunately, there are research data suggesting that the detection of psychiatric disorders by dermatologists is not completely satisfactory, and that dermatologists miss a substantial proportion of psychiatric pathology (8). Therefore, having an awareness of this rare condition will assist in the prevention of unnecessary investigation of such cases and will allow the early referral of patients for appropriate psychological counseling. Also, early diagnosis is important to prevent unnecessary drug therapy and chronic morbidity; by this way society can be protected from escalating and unnecessary expenditure of medical resources.
